# Methicillin-Resistant *Staphylococcus aureus* in Saarland, Germany: A Statewide Admission Prevalence Screening Study

**DOI:** 10.1371/journal.pone.0073876

**Published:** 2013-09-11

**Authors:** Mathias Herrmann, Christine Petit, Alik Dawson, Judith Biechele, Alexander Halfmann, Lutz von Müller, Stefan Gräber, Stefan Wagenpfeil, Renate Klein, Barbara Gärtner

**Affiliations:** 1 Institute and State Laboratory of Medical Microbiology and Hygiene, Saarland University Medical Center, Homburg, Germany; 2 Institute of Medical Biometry, Epidemiology, and Medical Informatics, University of Saarland, Homburg, Germany; 3 Saarland Ministry of Social Affairs, Health, Women, and Family, Saarbrücken, Germany; University of Buea, Cameroon

## Abstract

**Background:**

The screening of hospital admission patients for methicillin resistant *Staphylococcus aureus* (MRSA) is of undisputed value in controlling and reducing the overall MRSA burden; yet, a concerted parallel universal screening intervention throughout all hospitals of an entire German Federal State has not yet been performed.

**Methodology/Principal Findings:**

During a four-week period, all 24 acute care hospitals of the State of Saarland participated in admission prevalence screening. Overall, 436/20,027 screened patients revealed MRSA carrier status (prevalence, 2.2/100 patients) with geriatrics and intensive care departments associated with highest prevalence (7.6/100 and 6.3/100, respectively). Risk factor analysis among 17,975 admission patients yielded MRSA history (OR, 4.3; CI_95_ 2.7–6.8), a skin condition (OR, 3.2; CI_95_ 2.1–5.0), and/or an indwelling catheter (OR, 2.2; CI_95_ 1.4–3.5) among the leading risks. Hierarchical risk factor ascertainment of the six risk factors associated with highest odd’s ratios would require 31% of patients to be laboratory screened to allow for detection of 67% of all MRSA positive admission patients in the State.

**Conclusions/Significance:**

State-wide admission prevalence screening in conjunction with risk factor ascertainment yields important information on the distribution of the MRSA burden for hospitals, and allows for data-based decisions on local or institutional MRSA screening policies considering risk factor prevalence and expected MRSA identification rates.

## Introduction

Methicillin resistant *Staphylococcus aureus* (MRSA) is a major cause for healthcare associated infections (HAI), and considered a relevant patient safety issue. Infection control programs worldwide have proposed and implemented various strategies against the spread of this pathogen. Risk factors have been associated with MRSA carriage and invasive disease [1], [2], yet, it has become clear that risk factors for MRSA acquisition in the hospital have to be separated from those associated with patients already MRSA-positive upon hospital entry. Subsequently, MRSA screening and risk factor analyses on admission have been performed, risk factor scores for selective screening have been developed [3], and in some countries directly neighbouring Germany (e.g., the Netherlands and Denmark) such early-implemented targeted screening and isolation strategies (‘search and destroy’) have been successfully applied for controlling the MRSA epidemics [4], [5]. Moreover, in many countries with MRSA endemicity, national guidelines now recommend application of such risk factor-based screening as part of an ‘active detection and isolation’ (ADI) strategy [6]. Moreover, in a multifactorial approach analyzing the association of MRSA prevalence and key infection control parameters in 146 European hospitals, the implementation of an MRSA screening policy was found to be associated with decrease in MRSA prevalence [7]. As a result, in a consensus statement the European Society of Clinical Microbiology and Infectious Diseases (ESCMID) concluded that policies should be guided by local MRSA infection and colonization rates [8]. This consensus statement has recently been updated [9]. Despite intense research, guideline implementation, and policy making, the issue of an optimal cost-effective and patients safety-focussed approach towards admission screening remains debated as carefully performed studies have come to contrasting results [10]–[12]). While universal admission screening may most effectively prevent MRSA infections due to unrecognized transmission [13], [14], the presumed high costs associated with testing and contact precautions have also prevented its wide adoption [15].

This debate may be a consequence of the local/regional coverage of the screening policy in the various studies: An analysis applying extensive mathematical modeling confirmed previous observations, i.e. that admission screening will be less effective and more costly if neighboring hospitals do not screen [16]. Thus, it becomes clear that effective detection for MRSA has to be implemented with a strategy well beyond single departments or hospitals. The German Antibiotic Resistance Strategy (DART) [17] has addressed this issue by fostering regional German model projects for establishing regional networks on prevention and combat of antibiotic resistances and their spread. As part of this initiative, we have established the first State-wide German network for the control of MRSA, MR*SAar*Net (www.mrsaar.net).

The aim of this prospective cohort study was to define the MRSA and MSSA admission prevalence covering all hospitals in one German state. This evaluation was accompanied by an ascertainment of risk factors for MRSA acquisition. The overall goal was to provide a reliable database for establishing specific, region-tailored recommendations for an effective screening policy.

## Methods

As a study prerequisite, we intended to exclude any bias due to inter-laboratory or seasonal variabilities by processing all samples in one diagnostic laboratory (Institute of Medical Microbiology and Hygiene, University of Saarland Medical Center) during the period of October and November, 2010. Accordingly, during the study period an anticipated processing work load of 20.000–30.000 specimens with peak numbers of up to 1.500–2.000 specimens per workday were expected. Despite this elevated workload, a minimal turn-around time needed to be assured because participating hospitals expected the report of confirmed results within same time intervals as those offered by their routinely commissioned laboratories. This high number of specimen required the implementation of novel, automated methods for processing.

Accordingly, we opted for an automated sample processing system, the Walk Away Specimen Processor, WASP® (Copan, Brescia, Italy) [18], [19]. The use of WASP relies on a liquid based specimen microbiology technique, and we employed the flocked swab system provided by the WASP manufacturer allowing for immediate release of swabbed microorganisms into the Amies medium (ESwab™, Copan) without the need of a separate enrichment step. This technique has been evaluated for MRSA screening purposes [20], [21]. Specimens were directly plated on CHROMagar MRSA/CHROMagar *S. aureus* detection biplates (Mast, Germany). Prior to initiation of the main study, the plating protocol for WASP (using 30 µl of the Amies medium eluate) was optimized by careful comparison with the results of parallel manual streaking either of the ESwab or of 30 µl of Amies medium. The streaking pattern by WASP was found to be of particular importance, and a distribution of the 30 µl calibrated loop content through the entire length of the 80 mm CHROMagar biplate was found to be essential to achieve optimal results.

A prestudy was performed to assure that this automated liquid microbiology technique results in similar MRSA detection rates compared to conventional detection rates using cotton swabs and broth enrichment. The ESwab system was compared with conventional cotton swab specimen followed by enrichment (tryptic soy broth, 18 h, 35°C) at 195 admission patients of the Department of Urology, University of Saarland Medical Center, prospectively examined with parallel nasal swabs. The overall number of *S. aureus* (MRSA and MSSA) detected by either method was 66/195 (33.8%) specimen. Both methods identified the identical 7 MRSA among these 66 *S. aureus* isolates (10.6%)(overall MRSA prevalence 7/195 [3.6%]) while of the remaining 59 MSSA isolates, 21 were detected only by either method (with the cotton swab/enrichment method missing 8 and the ESwab/Amies method missing 13 isolates). Hence, the advantages of parallel testing in all Saarland hospitals, analysis in one centralized laboratory, and rapid communication of any positive result (*i.e.* features ascertained by the novel ESwab/WASP method) to our opinion clearly outweighed a potential slightly reduced MSSA detection sensitivity, and this method was selected as the standard procedure for the main study.

### Region

The State of Saarland is located in the southwest of Germany and is neighbored by France, Luxemburg, and the German State of Rhineland-Palatinate. It comprises of 1.03 millions inhabitants, and ∼250.000 patients are annually admitted to any of the 24 Saarland Hospitals (including one University hospital) with altogether 6800 beds (data from Ministry of Health and Consumer Protection, Saarland 2010).

### Participating Hopitals, Study Period

All 24 hospitals of the region participated in the study comprising of 12 hospitals with less than 200 beds, 10 hospitals with up to 500 beds and two hospitals with 600 and 1300 beds, respectively. 5.8% of the beds were located in intensive care units, 2.4% in geriatrics, 13.3% in psychiatry, and 3.3% in pediatrics; the remaining beds were located in various adult acute care departments. During a 4-week study period, participation was offered to 90–100% of the patients in 16 of the 24 hospitals. In 4 hospitals, 80–90% of the patients were asked for participation. Only in 4 hospitals, the participation was lower with rates of 79%, 61%, 38% and 34%, respectively.

### Patients

As sole inclusion criterion, admission to any of the 24 participating hospitals during the study period was employed. The only exclusion criterion was lack of written informed consent. In case of readmission, transfer to another hospital or admission to another hospital within the study period, a patient might have been included more than one time. Upon admission, each participating patient received a pseudonym identifier, allowing the hospitals to allocate positive MRSA results to patients.

### Questionnaire

Each patient or their legal representative was asked to fill out a risk factor evaluation form providing pseudonym identifiers. As patient-related data, gender and year of birth were recorded. In Germany, the Commission for Hospital Hygiene and Infection Prevention at the Robert-Koch-Institute (RKI)(Berlin) has issued a list of risk factors recommended for risk-adapted screening purposes [22] which delineate the targeted MRSA screening requirements for acute care facilities according to the German ‘Infection Protection Law’. The RKI list contains 5 independent risk factors whose presence should prompt targeted screening, plus 6 additional factors requiring screening only if at least two of these factors are present. These 11 risk factors were also evaluated in our study and were defined as follows: i) history of MRSA (subsequently referred to as ‘history’); ii) contact with an MRSA carrier (‘contact’); iii) skin ulcer, gangrene, chronic wounds or deep tissue infection (‘skin’); iv) burn injury (‘burn’), v) indwelling (Foley) catheter (‘catheter’), vi) end stage renal failure and dialysis (‘dialysis’), vii) antibiotic therapy within the last 6 month (‘antibiotics’); viii) requirement of chronic care (‘chronic care’), ix) resident in an elderly/chronic care facility (‘facility’), x) previous hospital admission (within a period of 12 months, and with >1 day duration) (‘hospital’); xi) occupational contact to farm animals (‘farm’). Moreover, 3 additional risk factors whose inclusion into our study was suggestive due to recent literature data were also evaluated: xii) diabetes mellitus (‘diabetes’), xiii) transfer from another hospital (‘transfer’), and xiv) occupational contact to meat or meat products (‘meat’),

### Samples

Custom made ESwab bundles each containing two flocked swabs and one test tube containing the modified liquid Amies medium (1 ml) were purchased (Copan). One of the swabs was used for the pharyngeal site and the other for both anterior nares. While the pharyngeal swab was swivelled in the elution medium then discarded, the nasal swab was immersed in the medium and remained until processing in the laboratory. In case of a patient with a breach of the intact skin, e.g. an i.v. catheter, a percutaneous epigastric tube or an open wound, maximal one additional swab from this site was sampled (decision on the sampling site at the discretion of the examiner considering highest suspicion for positivity). ESwab specimens labelled with pseudonym numbers were daily transferred to the laboratory and processed. All MRSA positive culture isolates were further confirmed using a penicillin binding-protein 2a latex agglutination test, and further subjected to *spa* typing as previously described [23].

### Ethics Statement

This study was approved by the Ethics Committee at the Chamber of Physicians of the State of Saarland (approval no. Nr. 127/10) and by the Saarland State Commissioner for Data Protection. All participants or their legal representative gave written informed consent.

### Statistical Methods

For categorical variables all figures are absolute or relative frequencies. For the comparison of age between the MRSA colonized group and the study population the t-test was used. Univariate risk analysis was performed with Chi-squared test or Fisher's Exact test. The multivariable risk analysis was performed with conditional logistic regression. Mean overall prevalence rates were calculated according to a random effects model to account for possible cluster effects. Any p-values given are two-sided and subject to a significance level of 0.05 resp. 95% confidence intervals. All analyses were done using IBM SPSS Version 21 and StatsDirect Version 2.7.9.

## Results

### Study Population

During the study period, 24,753 admissions were reported. Altogether, 20,690 (83.6%) patients were invited for participation. It is likely that there were various reasons for non-invitation (such as insufficient study information provided or involuntary neglect by staff). 663 (3.2%) patients either refused to participate, or informed consent could not be obtained, resulting in a number of 20,027 screened patients (overall participation rate, 83.6%)(cohort referred to as ‘patients’).

The parallel initiation of universal screening in all study hospitals, and the time interval required for corrective feedback between study center and admission personnel in the various hospitals contributed to the fact that specimens from 2,052 of these 20,027 patients were not accompanied by a fully evaluable questionnaire, and/or specimens were not labeled by a correct pseudonym identifier (also likely caused by the fact that the distributed information on the study including the requirement to return filled questionnaires may not have been available or overseen by part of the admission staff personnel). This resulted in a number of 17,975 patients (referred to as ‘study population’ and ‘study patients’) fully evaluable for the association of screening result and risk factor analysis. Of this group, 955 (5.3%) patients were transferred from other hospitals.

### *Staphylococcus aureus*, MRSA and MSSA Admission Prevalence

Altogether, 3,558/20,027, i.e. 17.8/100 patients were found to be positive in nares/pharynx and/or wounds for *S. aureus*. Of these, 436 were found to be positive for MRSA, corresponding to an overall on-admission prevalence (attack rate) of 2.2/100 patients.

The mean overall MRSA prevalence between participating hospitals was 2.4/100 (CI_95_, 2.0/100–2.9/100 [adjusted for possible hetergeneity between hospitals]; range 0/100 to 9.0/100). 3,122/20,027 patients carried MSSA corresponding to an overall MSSA prevalence of 15.6/100 admissions. Between participating hospitals, the mean adjusted MSSA prevalence was 20.0/100 patients (CI_95_, 16.0/100–23.0/100, range 6.9/100 to 32.9/100). The mean “proportion of resistant isolates” (MRSA/S. aureus) [24] between hospitals was 14.0%. This proportion corresponds to a ratio of approximately one MRSA positive patient for every 7 S. aureus positive patients.

### MRSA and MSSA Prevalence as a Function of Sample Site Localization

In 51/436 MRSA positive patients (11.7%) swabs from wounds, catheter exit sites or other lesions (as defined by the questionnaire) were tested as well; in one patient, only a swab from the wound was tested without accompanying nares/throat swab (resulting in 50 patients with samples from both areas). Of these 50 extranasal/oral specimen, 15 were found to be MRSA negative, 23 were positive both in nares/throat and in the extranasal/oral site, and 12 were positive only in the extranasal/oral site (and not in the nares/throat). This indicates that among MRSA positive patients which were also tested for MRSA colonization of extranasal/oral sites, 70% of these sites were found to be positive. Among patients with a positive MRSA result from a positive extranasal/oral site, 34% were found to be MRSA negative in their nares/throat.

With regard to MSSA, in 93/3,122 of MSSA positive patients, extranasal/oral sites were tested as well (3.0%). Of these extranasal/oral specimen, 27 were found to be MSSA negative, 44 were positive both in nares/throat and in the extranasal/oral site, and 20 were positive only in the extranasal/oral localization. This indicates that among MSSA positive patients also tested for colonization of extranasal/oral sites, 67.7% were found to be colonized or infected with MSSA in these sites. Similar to the results obtained for MRSA, around 1/3 of patients with MSSA positive extranasal sites were not colonized with these microorganisms in their nares/throat (31.8%).

### MRSA/MSSA Prevalence according to the Department of Admission

For 384/436 MRSA positive patients, a questionnaire with completed risk factor evaluation form was available. 22/384 (5.7%) patients were transferred from another hospital. A valid indication of the clinical department upon admission was available for 361/384 forms, and this cohort was analyzed with respect to the attribution of MRSA admission rates to the respective medical specialty.

Admission to a geriatric department was associated with the highest MRSA admission prevalence, followed by intensive care units. Admission to a general medical or surgical department was associated with MRSA prevalence close to the mean prevalence found in our study, while admission to a psychiatric and pediatric department was associated with the lowest prevalence (Table 1).

**Table 1:** MRSA admission prevalence according to medical specialty.

Medical Specialty	MRSA detected (n)	Admission patients screened (n)	Admission prevalence(MRSA/100 patients)
Geriatrics	17	225	7.6
Intensive Care	17	269	6.3
Internal Medicine	142	4,885	2.9
Neurology	20	866	2.3
Heart and Thoracic Surgery	95	4,507	2.1
Orthopedics	18	967	1.9
Urology	12	678	1.8
Dermatology	5	299	1.7
Gynecology/Obstetrics	14	881	1.6
Radiotherapy & nuclear medicine	3	212	1.4
Eye	4	357	1.1
Ears-Nose-Throat	9	955	0.9
Psychiatry	1	138	0.7
Pediatrics	4	660	0.6
Unknown/Others	23	2,076	1.1
Total	384	17,975	2.2

### *S. aureus* Protein A (spa) Typing

A total of 382/436 MRSA isolates was available for *spa* typing. 225 (58.9%) of these isolates were attributable to *spa* sequence type t003 (corresponding to ST5/CC5, the Rhine-Hesse/EMRSA-3/New York clone), 62 (16.2%) to t504 (closely related to t003), 14 (3.7%) to t002 (ST5/CC5, related to t003), and 9 (2.4%) to t008 (ST-8/CC8, Northern German MRSA, USA300 cMRSA). 5 isolates were attributable to t032 (ST-22, Barnim MRSA, EMRSA-15), t045 (ST-5), or t458, respectively. Another 46 *spa* types occurred at a rate of <1%. One isolate revealed *spa* type t011 (ST-398, livestock-associated*)(spa*-MLST mapping according to http://spa.ridom.de/mlst.shtml, February 08, 2013).

### Age and Gender

**Figure 1:**
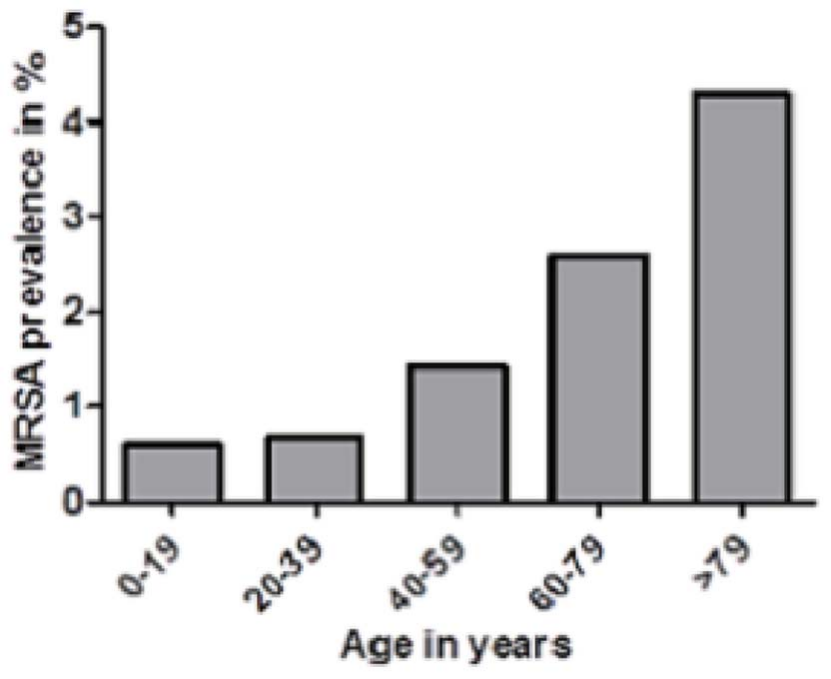
MRSA prevalence as a function of age.

For 17,244/17,975 patients of the study population, the date of birth was available. MRSA carrier status was demonstrated in 373 of these 17,244 patients. The mean age of the study population was 56.5 years (SD: 12.3 years), the median age was 63 years (25^th^ and 75^th^ percentile, 45 and 76 years, respectively). MRSA colonized study patients were significantly older than the overall study population (mean age, 67.5 years [SD: 36.0], median 76 years [25^th^ and 75^th^ percentile, 64 and 84 years, respectively]) (*p*<0.001)(for details in age related prevalence please refer to Figure 1). For 17,415/17,975 study patients, valid gender identification was available; 8333 of these patients (47.9%) were males. MRSA prevalence was significantly higher in males than in females (207/8333 [2.48%] vs. 171/9082 [1.88%]; *p* = 0.007).

### Risk Factor Analysis

Only 40/384 (10.4%) of all MRSA colonized study patients did not report any risk factor. The distribution of risk factors in MRSA positive study patients is shown in Table 2. Univariate analysis revealed that 10 out of 14 risk factors evaluated were found to be associated with a significantly increased MRSA positivity; only ’farm’, ’meat’, ‘burn’ and ‘transfer’ were not significantly associated with MRSA carriage upon admission (Table 2).

**Table 2:** Univariate analysis of risk factors associated with MRSA carriership upon admission and conditional logistic regression (only significant risk factors depicted in the table).

Risk factor (abbreviated)	Univariate analysis			Conditional logistic regression analysis	
	n/N	OR	95% CI	OR	95% CI
history	450/17,975	9.9*	7.5; 13.1	4.3	2.7; 6.8
skin	816/17,279	6.9*	5.3; 8.9	3.2	2.1; 5.0
chronic care	1,615/17,355	6.4*	5.1; 7.9	1.8	1.2; 2.9
facility	813/17,222	4.8*	3.7; 6.4		
dialysis	169/17,401	4.3*	2.5; 7.5		
catheter	1,199/17,453	3.5*	2.7; 4.6	2.2	1.4; 3.5
contact	790/13,276	3.3*	2.3; 4.7	1.8	1.2; 2.9
diabetes	2,847/16,956	2.5*	2.0; 3.1	1.9	1.4; 2.7
hospital	7,702/16,940	2.2*	1.8; 2.8	1.5	1.1; 2.0
antibiotics	5,179/15,760	2.2*	1.8; 2.8		
transfer	955/17,975	1.1	0.7; 1.7		
burn	69/17,439	0.7	0.1; 5.0		
farm	287/17,357	0.5	0.2; 1.5		
meat	291/17,348	0.3	0.1; 1.3		

Risk factors are listed according to OR values of univariate analysis. For detailed description of the risk factors ascertained, please refer to Methods.

* = P<0.05.

In contrast, in a multivariate analysis using conditional logistic regression, only 7 factors were significantly associated with an increased risk: ’history’, ‘skin’, ‘catheter’, ‘diabetes’, ’chronic care’, ‘contact’, and ‘hospital’ (Table 2).

With respect to the relative risk for MRSA carriage of the various factors evaluated as a function of age, we selected the age of 40 years and above as cutoff (the MRSA prevalence in the patient group younger than 40 years was <1/100), and analyzed risk factors in this subpopulation. It appears that in this elder population, the multivariate ORs are very similar to the ORs in the entire study group (data not shown).

### Risk Factor-based Number Needed to Screen and Resulting MRSA Detection Fractions

Risk factors were sorted according to the rank of significant OR results in the multivariate analysis, beginning with the risk factor with highest OR (Table 3). First, the absolute number of risk factor entries in the study population (n = 17,975) was ascertained. Next, upon hierarchical database retrieval beginning with the risk factor associated with highest OR (‘history’), the number of cumulative risk factor entries was assessed, and expressed in percent of the study population. This percentage indicates the proportion of admission patients to be screened. Correspondingly, the number of MRSA detected (as a function of risk factor entry) was determined, both in absolute as well as in cumulative numbers. The percentage indicates the proportion of MRSA detected upon risk factor based screening. For instance, if only the top 3 risk factors (‘history’, ‘skin’, 'catheter') are ascertained, 11.9% of admission patients need to be screened resulting in the detection of 41% of all MRSA positive patients.

**Table 3:** Cumulative proportions of patients reporting one or several risk factors and resulting number-to-screen (applying risk factors significant in conditional logistic regression analysis, see Table 2).

Risk factor (abbreviated)	Risk factor entries	MRSA detected
history	450 (2.5%)	69 (17.9%)
history+skin	1,158 (6.4%)	119 (30.9%)
history+skin+catheter	2,141 (11.9%)	158 (41.1%)
history+skin+catheter+diabetes	4,373 (24.3%)	204 (53.1%)
history+skin+catheter+diabetes+chronic care	5,037 (28.0%)	246 (64.0%)
history+skin+catheter+diabetes+chronic care+contact	5,558 (30.9%)	256 (66.7%)
history+skin+catheter+diabetes+chronic care+contact+hospital	10,132 (65.4%)	320 (83.3%)
Study population	17,975	384

If patients are screened with respect to any of the first 6 risk factors in multivariate analysis significantly associated with MRSA carriership, 30.9% of patients would be tested to identify 66.7% of MRSA positive patients. In particular, the inclusion of ‘hospital’ as the least-ranked yet most frequently entered significant risk factor requires the screening of an additional 34.5% of patients (in our study, 4,574 patients) but reveals only an additional 16.6% of MRSA positive patients (64 patients).

In addition, it was of interest in our study to compare the results when employing the list of risk factors established by RKI with the risk factors determined in our study. In line with the RKI recommendations the following risk factors were analyzed: five risk factors (‘history’, ‘facility’, ‘hospital’, ‘farms’, ‘contact’) independently prompting an indication to screening plus six factors considering a risk only if at least two of these factors are present (‘chronic care’, ‘antibiotics’, ‘catheter’, ‘dialysis’, ‘skin’, ‘burn’). Overall, employing the RKI risk criteria, 9,099/17,975 (50.6%) patients would be needed to be screened conferring a detection rate of 298/384 (77.6%) of MRSA.

### Cost – Benefit Estimates of a Screening Program in Saarland

Under these assumptions we are now able to define the MRSA burden for Saarland hospitals. Given the annual number of hospital admission in Saarland and the MRSA admission prevalence, we can robustly determine the number of MRSA admissions in the State of Saarland to be ∼5000/annum. This figure raised, it is now also possible to roughly estimate the direct costs associated with the implementation of targeted, risk factor-adapted screening. Laboratory costs may vary by the laboratory-specific diagnostic procedure, but employing combined conventional culture using selective media it can be reasonably estimated as approximately 5 €/assay. If a targeted screening approach is selected, the personnel and infrastructure costs for risk factor ascertainment also need to be accounted for. Accordingly, screening between 30% and 40% of admission patients would amount to expenses between 0.5–1.0 million € annually in Saarland. On the other hand, it has been estimated that the rate of infection after MRSA colonization ranges between 11% and 36% [13], [25]–[27]). Giving a conservative estimate for an attributable excess costs for MRSA infection (a controversially discussed figure in the literature [28]–[30]) in the order of 10.000€ per infection, the annual costs of these infections would be in the order of 10–15 million €. While such considerations can not substitute for a clear-cut cost-benefit calculation, in context with information on the overall and institution-specific MRSA carrier rates they could be shared with the participating Saarland hospital administrators in order to allow for a hospital based, individual analysis and decision on the future screening policy.

## Discussion

The here presented large study analyzing the MRSA admission prevalence in an entire German Federal State has been carried out according to a unique study concept of identical preanalytical and analytical detection methods performed in a single laboratory during a short parallel study period of four weeks, in order to minimize bias by external factors. Such a parallel, prospective universal admission screening approach has only rarely been undertaken elsewhere; rather, in the literature, most admission screening studies were restricted to single or few hospitals often including only a few medical departments or smaller areas, and employing various culture based and molecular detection techniques.

Data from published studies allow to compare the admission prevalence of 2.2/100 patients determined in our study with the situation elsewhere. A non-exhaustive list from these reports from European countries [11], [31]–[38] and from North America [10], [25] reveals admission prevalence values ranging between 3/100 and 9/100. One of the largest MRSA intervention trials, the Veterans Affairs Initiative to prevent MRSA infections [39] reported admission prevalence values as high as 13.6/100. In German hospitals, an MRSA point prevalence or on-admission prevalence between 0.7/100 and 5.3/100 [40], [41] was reported (of note, the hospital admission/discharge rates reported by the European Union for Germany are among the highest in the Union with 240 discharges/1000 population, with a mean among 24 EU countries of 176/1000 [42]; this fact underlines the need to collect local data as a basis for targeted screening particularly in high hospital admission-rate regions such as in Germany). Two larger studies, the Euregio study in Münsterland, and the study in Siegen-Wittgenstein should be mentioned separately: The Euregio study revealed an admission positivity rate of 1.6/100 admissions (total patient number, n = 25,540) [43]; yet, analyses were carried out in different testing laboratories. The Siegen-Wittgenstein study reported 1.4/100 admission MRSA prevalence (total patient number, n = 6,985) (*S. aureus* 22,3%) with the latter being performed both in acute care and rehabilitation facilities [44]. One recent study performed in one hospital in the State of Brandenburg, Germany, reported 0.8/100 MRSA prevalence (total patient number, n = 13.855) at admission [45]. These literature data demonstrate that it is almost impossible to extrapolate secular national supra-regional MRSA prevalence data on the MRSA burden for individual hospitals or regions. However, it is clear that solely based on clinical-microbiological cultures the prevalence of MRSA is grossly underestimated with 85% of MRSA-colonized patients being missed [46]. Hence, routine performance of at least targeted MRSA hospital admission screening is now recommended [8], and has also been included in a number of health system and legislator policies. The scope of screening (universal versus targeted) as well as the method (molecular versus culture-based screening), however, depends on the baseline on-admission prevalence as summarized in a recent review [9].

Yet, how should a screening program be introduced for a given hospital or region, if such robust baseline data are not available? To respond to this question, we initiated this study in order to provide hospitals and State policy makers with the necessary data on the MRSA admission burden for the State of Saarland hospitals. As a prerequisite to convince the directors of all hospitals to collaborate in this universal screening approach, the time frame of the study needed to be limited to a maximally 4-week-screening period. Moreover, in a system with hospitals typically employing diagnostic services from different laboratories (including different microbiologic-analytical protocols), subtle but potentially important differences in the MRSA prevalence were expected. Thus, to overcome this problem, a single laboratory was selected to process all samples. In addition, due to the short and parallel time frame, the large number of daily analyses required automation in specimen handling, a prerequisite being met with the automated specimen processor employed.

Our study clarified a number of other important issues. While we observed a variable MRSA admission prevalence between the institutions, the overall differences were minor with exception of four hospitals with MRSA rates above 4.0/100 admissions (these latter receiving a larger number of elderly, gerontopsychiatric patients)(data not shown). In accordance with findings in the Euregio [43], the MRSA admission prevalence in 16/24 Saarland hospitals was found to be between 1/100 and 3/100 patients. Between various departments, important differences were noted, and geriatric departments as well as ICU’s in Saarland reported the highest MRSA admission prevalence putatively due to differences in hygiene, antibiotic prescription and disease severity issues [11], [25]. Elevated admission prevalence (2.6/100) in both geriatric/rehabilitative and ICU wards (when compared to the mean prevalence of 1.4/100) was also reported in the Euregio study [43], yet this was clearly inferior to the prevalence observed in the respective departments in Saarland hospitals.

The Saarland study population also revealed relevant details with respect to the risk factors significant for MRSA carriage. Interestingly, risk factors with highest OR’s in our analysis as well as in the Euregio study were MRSA ‘history’ and a ‘skin’ condition, yet, in our study the associated relative risks were lower when compared to Euregio (with odd’s ratios of these two factors reported of 67 and 55, respectively). In the Euregio study, residency in a care institution (‘facility’) proved also to be highly associated MRSA carrier status (significant in our study only in univariate analysis), while ‘catheter’ as a medical condition was again relevant both in the Euregio as well as in the Saarland study. The other medical conditions significant in our study, ‘diabetes’, was not ascertained in the Euregio study, yet, ‘contact’ with an MRSA carrier was a significant factor in both studies. Moreover, in both studies, previous hospitalization (‘hospital’) was also a significant albeit weak risk factor, yet, it is reported by many admission patients, and its inclusion in a targeted screening algorithm greatly augments the number-to-screen. Interestingly, the use of antibiotics in the past 6 months (‘antibiotics’) – a significant risk factor in other studies [11] – has not been found to be a significant risk factor in multivariate analysis in either the Saarland or the Euregio study. Other factors such as occupational contact with meat products or contact with farm animals were not found to be relevant in the Saarland region; this is also in line with the finding of only one isolate attributable to the t011/ST398 genotype and typically associated with livestock-associated MRSA (this might be mostly due to the fact that economically this region is dominated by metallurgy and other industry, and pig farming associated with livestock MRSA is of negligible importance in this area).

This study has potential limitations. The relatively low prevalence of *S. aureus* in our study population raises the question on the sensitivity of our screening and test system. The major methodological difference to all other previous studies is the consequent use the flocked ESwabs [20], [21] in our study, precluding the enrichment step used in other studies [43], [47]. The pre-study we performed yielded *S. aureus* admission screening rates corresponding to previously reported rates [25], [43], [44], and being comparable between the direct ESwab chromogenic plating method and the enrichment of conventional swabs (of note, some MSSA positive samples were missed by the conventional method with cotton swabs either). Ultimately, the reason for the lower prevalence of *S. aureus* in the main study remains unclear, and can not easily be attributed to the quality of sampling (given written and web-based instructions on the correct swabbing technique and a great deal of enthusiasm and support for this study in the participating hospitals). Another limitation of our study may concern the quality of risk factor evaluation which was not controlled for, and might have been underreported particularly for risk factors whose ascertainment depends on proper evaluation of patient history (e.g. ’antibiotics’). Finally, it should be mentioned that the laboratory diagnostic concept of our study, i.e. performing all tests in a single centralized laboratory staffed 7/7 days, and responding on a rapid schedule, may not necessarily correspond to a ‘true life’ experience in other regions or institutions served by centralized microbiologic laboratory facilities involving extended transport and storage times.

On the other hand, our study is a ‘true life’ experience in that risk factor evaluation was obtained by the same personnel which will subsequently identify admission patients for targeted screening. Therefore, a clear limitation of the number of risk factors prior to decision for or against routine screening is urgently needed. The German recommendations for screening of MRSA admission patients [22] with altogether 11 risk factors, several of them difficult to evaluate, and 6 of them only to be applied if reported in combination may overstrain the hospital personnel’s capacities especially during the admission process. As a consequence of our study, we recommended the participating institutions to develop their own risk attributed screening policy based on the results of our analysis; yet, as a ‘default strategy’ we suggest to employ the six factors with highest and significant risk whose ascertainment and subsequent screening of less than one third of Saarland admission patients would allow for identification of more than two third of MRSA cases. This set of risk factors also appears appealing as all these six factors – with exception of ‘contact’ – can be ascertained in the admission ward based on readily available medical and social information (‘chronic care’, ‘diabetes’), hospital MRSA alert systems (‘history’), and simple inspection of the patient (‘skin’ and ‘catheter’).

In conclusion, in this prospective admission prevalence study, the prevalence of MRSA, and thus the epidemiologic burden by patients colonized upon entry into acute care hospitals could be determined for an entire German federal state of Germany. In conjunction with an analysis of the absolute and relative importance of ascertained risk factors, such information provided a rational basis for participating hospitals to develop an institution-individualized policy for targeted or universal screening. Moreover – and probably as importantly albeit difficult to evaluate – this prevalence screening has raised the overall awareness among administrators and hospital employees of participating institutions as well as of local policy makers and the general public to control and prevent colonization and infection with this notorious nosocomial pathogen. This intervention is part of an ongoing network activity in Saarland involving hospitals and community-based institutions to reduce the burden of MRSA and other multiresistant pathogens.
